# Emerging Roles for LGR4 in Organ Development, Energy Metabolism and Carcinogenesis

**DOI:** 10.3389/fgene.2021.728827

**Published:** 2022-01-24

**Authors:** Linlin Yang, Jing Wang, Xiaodi Gong, Qiong Fan, Xiaoming Yang, Yunxia Cui, Xiaoyan Gao, Lijuan Li, Xiao Sun, Yuhong Li, Yudong Wang

**Affiliations:** ^1^ Department of Gynecological Oncology, The International Peace Maternity and Child Health Hospital, School of Medicine, Shanghai Jiao Tong University, Shanghai, China; ^2^ Shanghai Municipal Key Clinical Specialty, Shanghai, China; ^3^ Shanghai Key Laboratory of Embryo Original Disease, Shanghai, China

**Keywords:** LGR4, development, immunity, metabolism, malignancy, targeted therapy

## Abstract

The leucine-rich repeats containing G protein-coupled receptor 4 (LGR4) belonging to G protein-coupled receptors (GPCRs) family, had various regulatory roles at multiple cellular types and numerous targeting sites, and aberrant LGR4 signaling played crucial roles in diseases and carcinogenesis. On the basis of these facts, LGR4 may become an appealing therapeutic target for the treatment of diseases and tumors. However, a comprehensive investigation of its functions and applications was still lacking. Hence, this paper provided an overview of the molecular characteristics and signaling mechanisms of LGR4, its involvement in multiple organ development and participation in the modulation of immunology related diseases, metabolic diseases, and oxidative stress damage along with cancer progression. Given that GPCRs accounted for almost a third of current clinical drug targets, the in-depth understanding of the sophisticated connections of LGR4 and its ligands would not only enrich their regulatory networks, but also shed new light on designing novel molecular targeted drugs and small molecule blockers for revolutionizing the treatment of various diseases and tumors.

## Introduction

The leucine-rich repeat-containing G protein-coupled receptors (LGRs) are highly conserved proteins of the G protein-coupled receptors (GPCRs) family, identified as multiple repeats of leucine-rich repeats (LRRs) in the extracellular domain (Luo and Hsueh, 2006). The leucines in LRRs act as the dominant hydrophobic residue and play a critical role in the interactions between proteins (Kobe and Deisenhofer, 1993; 1995), which also allow forming a unique tertiary structure (Kajava, 1998). LRRs are connected to a seven-transmembrane (TM) domain that are related to G protein activation via a cysteine-rich region (Xu et al., 2013) and exerts its biological effects by binding to the ligand (Wang D. et al., 2013). LGRs could be subdivided into three groups (groups A–C) (Barker et al., 2013). Group A is consisted of lutenizing hormone receptor (LHR), follicle-stimulating hormone receptor (FSHR) and thyroid stimulating hormone receptor (TSHR) (Van Loy et al., 2008). Group B contains LGR4, LGR5, and LGR6, which exhibit a high degree of homology and function as receptors for the Wnt-activating R-spondins (Dubey et al., 2020). Group C is consisted of LGR7 and LGR8, which recognize relaxin (Yoshino et al., 2020) and insulin-like peptide 3 (INSL3) (Yeom et al., 2021), respectively.

The leucine-rich repeat-containing GPCR 4 (also called as LGR4) molecule is 107 kb and located on chromosome 11 at position 11p14-p13. It is composed of 17 LRRs and each contains 24 amino acids (McDonald et al., 1998). LGR4 signaling plays a functional role in self-renewal of stem cells by binding to R-spondin, thus potentiating Wnt signaling. R-spondin interacts with LGR4 inhibiting the expression of ZNRF3 and RNF43, the negative mediators of Wnt signaling that induce degradation of the Wnt receptor Frz and coreceptors LRP5/6 (Hao et al., 2012), thereby elevating the concentration of Wnt receptors and increasing the signaling response. R-spondin-bound LGR4 could also bind directly to LRP6 to boost the phosphorylation of LRP6 in response to Wnt-Fzd combination (Carmon et al., 2011). Clathrin (Glinka et al., 2011) and Norrin (Deng et al., 2013) were also reported to be needed for LGR4-mediated Wnt signaling. The ligand activated LGR4 triggers G-protein through GTP binding as well. Then coupled Gαs is dissociated from LGR4 and activates adenylyl cyclase (AC) elevating the level of second messenger cyclic AMP (cAMP), which activates protein kinase A (PKA) and in turn, phosphorylates cre-binding protein (CREB), thus enhancing the expression of its target genes, such as mineralocorticoid receptor (Wang et al., 2012), estrogen receptor α (Li et al., 2010). However, the ligands initiating cAMP/PKA pathway by LGR4 still remains unidentified.

Accumulating evidence supported by recent studies has shown that LGR4 is indispensable in embryonic growth, multiple organ development (Knight and Hankenson, 2014), energy metabolism (Li et al., 2014), ischemia/reperfusion injury (Li Z. et al., 2019) and the maintenance of stem cell self-renewal in intestine (Mustata et al., 2011), prostate (Luo et al., 2013), and mammary gland (Wang Y. et al., 2013). LGR4, as a new RANKL receptor, could counteract RANKL-driven osteoclastogenesis and enhance osteoblast maturation, mineralization (Luo et al., 2016) and vascular calcifcation (Carrillo-López et al., 2020). It also plays an oncogenic role in various human cancers, such as multiple myeloma (van Andel et al., 2017), thyroid carcinoma (Kang et al., 2017), and ovarian cancer (Wang Z. et al., 2020), etc. This paper will systematically summarize LGR4’s role in organ development, energy metabolism and tumor formation, which may provide the fundamental basis for the targeted gene therapy in the future.

## Methods

We screened MEDLINE, PubMed, and Google Scholar for relevant literatures from 2000 to 2021 and subjected the corresponding references to this review. This compiling was limited to studies written in English by using the terms “Leucine-rich repeats containing G protein-coupled receptor 4”, “LGR4”, “GPR48”, focusing on the biological function of LGR4, and various roles of LGR4 in development, immunity, energy metabolism, oxidative stress, and carcinogenesis.

## Signal Transduction of LGR4 Gene in Cells

**FIGURE 1:**
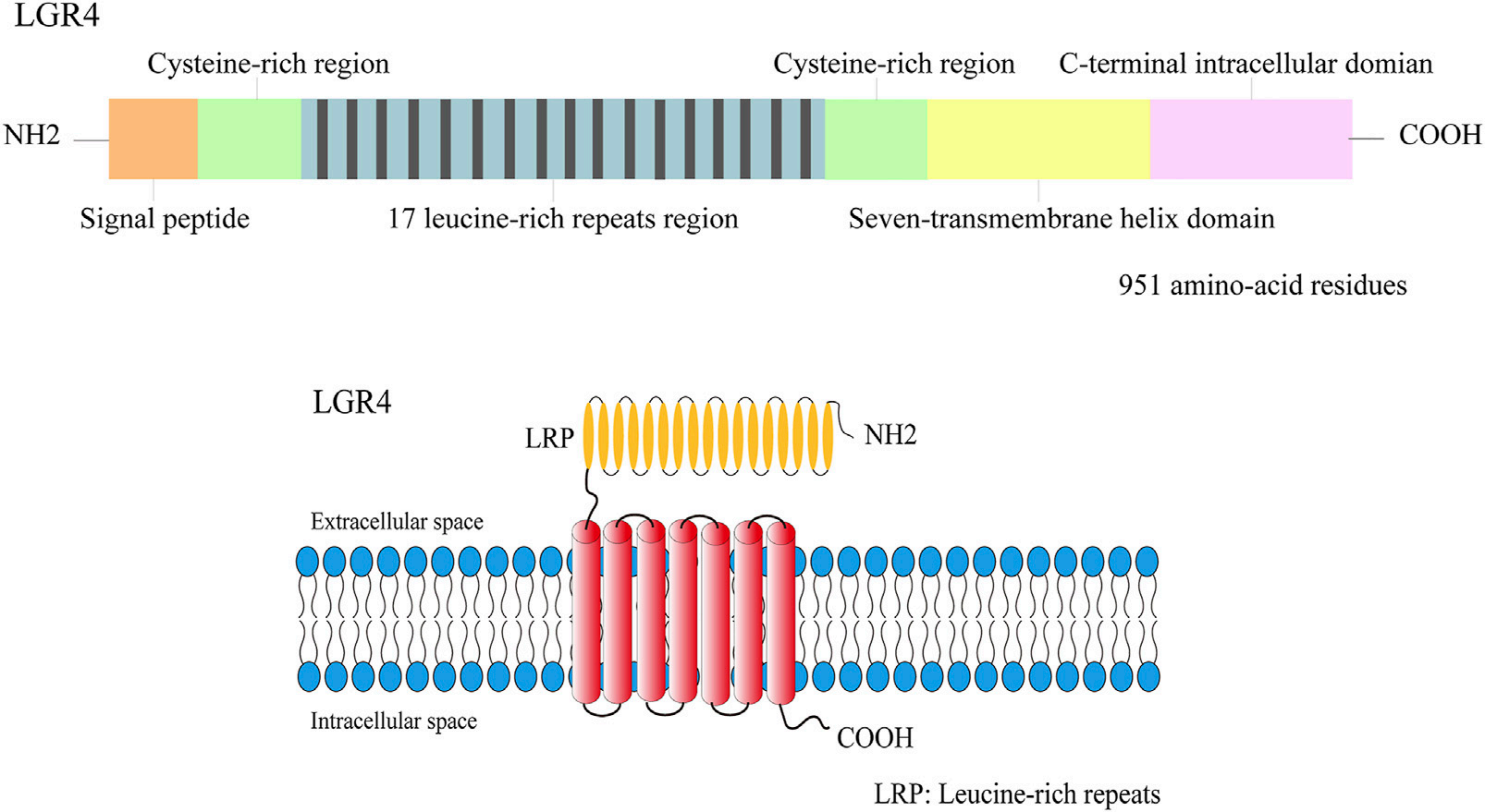
The structure illustration of the LGR4 protein. LGR4 was a member of transmembrane receptor, its N-terminal domain was comprised of 17 leucine-rich repeats region which was flanked by the N-/C- cysteine-rich regions. A seven-transmembrane domain and a C-terminal intracellular region were detected in LGR4.

**FIGURE 2:**
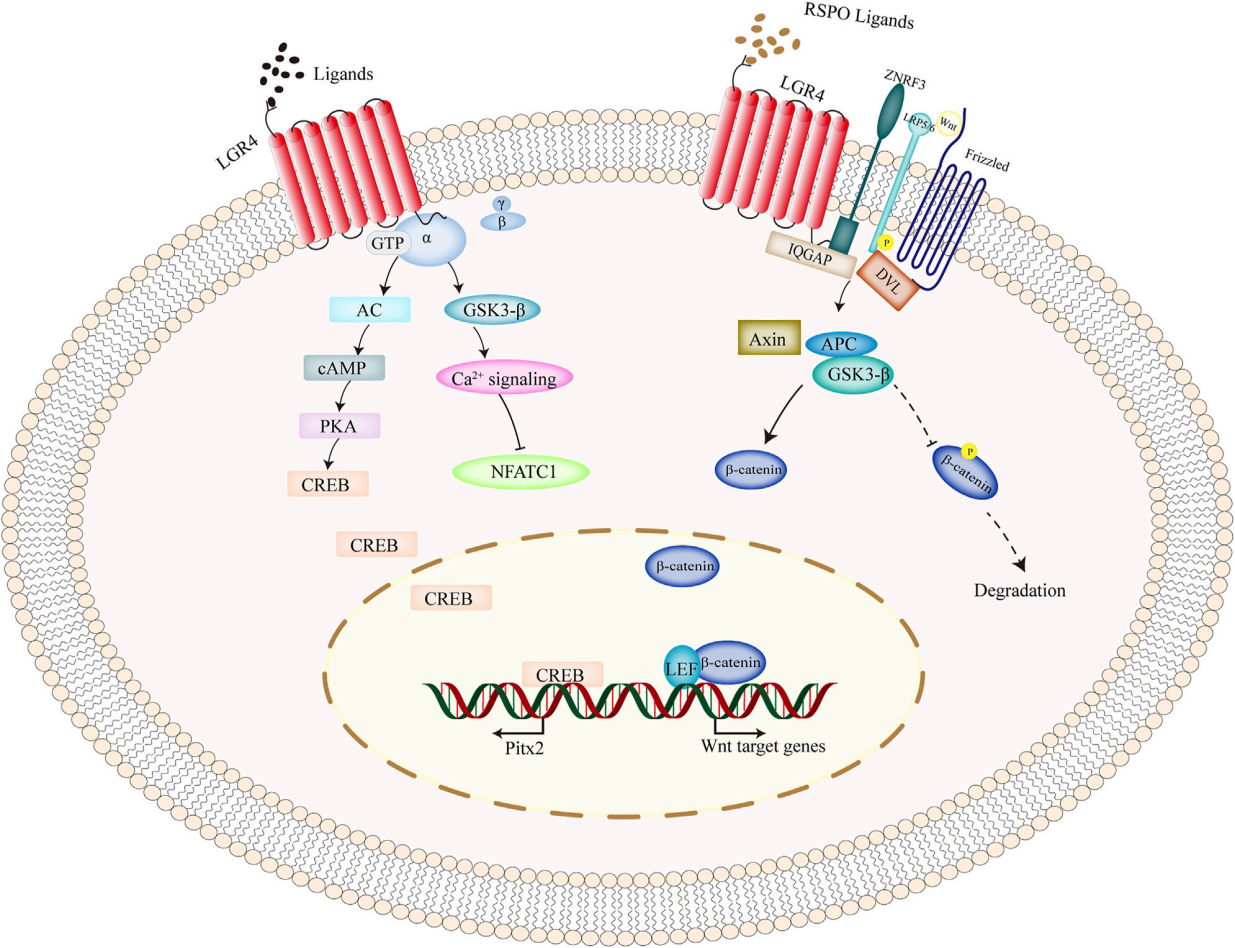
Intracellular signaling pathways of LGR4 gene. As a classical GPCRs molecule, upon ligands binding to LGR4, and it activated heterotrimeric G-proteins to transduce the cytoplasmic signal. Moreover, upon the stimulation of RSPO ligands, simultaneous binding of ZNRF3 and LGR4 suppressed the ubiquitination of frizzled receptor, promoted LRP phosphorylation, recruited IQGAP1 and increased its interaction with DVL, and thus activating the canonical Wnt signaling. β-catenin was prevented from degradation, translocated into the nucleus, and interacted with the transcription factors of TCF/LEF to induce the transcription of its target genes. Abbreviations: GPCRs, G-protein-coupled receptors; LGR4, leucine-rich repeat-containing G protein-coupled receptor 4; RSPO, R-spondin.

LGR4 is a transmembrane receptor of the GPCRs superfamily that is characterized by a large extracellular Leucine-rich domain that recognizes and interacts with its ligands (Van Loy et al., 2008), thus regulating numerous cellular processes (Figure 1). Many studies have explored the mechanisms of LGR4 gene. Researchers demonstrated that LGR, R-spondin, and ZNRF3 or RNF43 formed a ternary complex (Hao et al., 2012; Koo et al., 2012), alleviating ZNRF3/RNF43 clearance of Frizzled-LRP Wnt coreceptor, thus activating Wnt signaling (Hao et al., 2012). Rspos-LGRs signaling is essential for embryogenesis and cell protection (Knight and Hankenson, 2014). Another study found that RSPO-LGR4-IQGAP1 promoted MEK1/2-modulated phosphorylation of LRP5/6 in β-catenin-dependent manner or regulated actin dynamics in β-catenin-independent way, thus potentiating Wnt signaling (Carmon et al., 2014). Wang, D. et al. showed that the furin-like cysteine-rich domains of RSPO1 could interact with LGR4, thus inducing its biological activities (Wang D. et al., 2013). In addition, Park, S. et al. explored that full-length LGR4 interacted with E3 ligases RNF43 and ZNRF3 forming a complex to reduce ubiquitylation degradation of Wnt receptors and activate Wnt/β-catenin signaling (Park et al., 2020). They also explored that RSPO2 activated Wnt/β-catenin signaling with no binding to LGR4 or LGR5 (Park et al., 2018), which differentiated from other RSPO molecules (Figure 2). Moreover, Geng, A. et al. demonstrated that Rspo1 interacted with LGR4 and then activated cAMP-PKA signaling to elevate Esr1 expression and increase mammary side branches in a Wnt-independent manner (Geng et al., 2020), which provided a novel mechanism for estrogen-related diseases.

## LGR4 in Organ Development

### The Role of LGR4 Gene in the Development of Maxillofacial Organs

The gene of LGR4 was widely expressed in adrenal gland, kidney, heart, stomach, intestine, bone/cartilage and other tissues, and was first found to be associated with developmental processes (Yi et al., 2013), which was validated by immunohistochemical staining in wild-type mice. LGR4 deletion led to the severe pre- and postnatal lethality of mice explaining the significant importance of the LGR4 signaling for cell survival and growth (Mazerbourg et al., 2004). Meanwhile, LGR4 deficiency attenuated the expression of ATF4 via cAMP-PKA-CREB pathway to modulate definitive erythropoiesis (Song et al., 2008). Furthermore, LGR4 knockdown blocked GnRH neuron development by impairing Wnt/β-catenin signaling, leading to delayed puberty (DP) (Mancini et al., 2020). In addition, LGR4 regulated long term depression (LTD) at parallel fiber-PC (PF-PC) by modulating Creb signaling, suggesting its role in cerebellar ataxia (Guan et al., 2014). As a marker for hair follicle stem cell (Kim et al., 2019), LGR4 promoted the hair cycle progression by activating Akt/mTOR signaling, Wnt/β-catenin signaling and decreasing BMP signaling, thus regulating the development of hair follicle (HF). The skin epithelia-specific deletion of LGR4 lead to reduced numbers of LGR5^+^, and actively proliferating HF stem cells without affecting the number of quiescent HF stem cells, resulting in compromised HF regeneration after transplantation (Ren et al., 2020). It also played a critical role in controlling hair cell differentiation in cochlea (Żak et al., 2016). In eye development, Jin, C. et al. showed that LGR4 was highly expressed in cells of eyelids. LGR4 deficiency inhibited the phosphorylation of EGFR, thus blocking epithelial cell proliferation and migration in eyelid development (Jin et al., 2008). Additionally, LGR4 regulated keratinocyte proliferation through EGFR signaling pathway and the inhibitor of EGFR tyrosine kinase or its ligand HB-EGF could suppress cellular processes (Wang et al., 2010). The keratinocyte motility was reduced in LGR4 deleting mice leading to eye-open at birth (EOB) phenotype (Kato et al., 2007). Weng, J. et al. showed that deletion of LGR4 downregulated Pitx2 via cAMP-CREB signaling, thus inducing ocular anterior segment dysgenesis (ASD) (Weng et al., 2008). Further, the antioxidant enzymes CAT and SOD1 were downregulated in the lens epithelial cells of LGR4 deficiency mice, resulting in cataract formation (Zhu et al., 2015). MicroRNA let-7b promoted the apoptosis of lens epithelial cell by targeting LGR4, thus inducing age-related cataract (Dong et al., 2016). LGR4 could also be directly targeted by miR-34a and the downregulation of LGR4 inhibited the proliferation, migration of retinal pigment epithelial cell (Hou et al., 2016). LGR4 inactivation decreased histone demethylases Jmjd2a and Fbxl10 via cAMP-CREB signaling, thereby reducing the expression of development-related genes and increasing cell apoptosis, resulting in aniridia-mental retardation syndrome (Yi et al., 2014). Intriguingly, RSPO2 cooperated with WNT9b potentiating WNT/β-catenin signaling to regulate mouse facial development, while Jin, Y. R. et al. reported that LGR4/5/6 receptors played less critical roles in their cooperation to control facial development (Jin et al., 2020). Consistently, Szenker-Ravi, E. et al. explored that RSPO2, without the interaction with LGR4/5/6 receptors, served as a direct antagonistic to the ligases of RNF43 and ZNRF3, which together governed limb development (Szenker-Ravi et al., 2018). Then more relevant studies were needed to validate this specific mechanism.

### LGR4 in Bone Differentiation and Mineralization

LGR4 was believed to be a novel receptor for RANKL, it could induce the cAMP-PKA-CREB signaling to control the expression of Atf4 and its target genes Ocn, Bsp and collagen in osteoblasts. LGR4 deficiency in murine led to a delay in osteoblast differentiation, while increasing the activity of osteoclasts, thus regulating bone remodeling (Luo et al., 2009). LGR4 could also compete with the canonical receptor RANK to bind RANKL, suppress RANKL-RANK-TRAF6 signaling cascade and activate the Gαq and GSK3-β signaling, thus inhibiting the activity of NFATC1 and blocking RANKL-induced osteoclast differentiation (Luo et al., 2016). Jang, Y. et al. identified that the mutated RANKL protein acted as a competitive inhibitor of RANKL, bound only to the receptor LGR4, induced GSK-3β phosphorylation and inhibited NFATc1 nuclear translocation, and thereby preventing osteoclast differentiation (Jang et al., 2021). Additionally, miR-34c promoted osteoclast differentiation through targeting LGR4, activating NF-κB and GSK3-β signaling (Cong et al., 2017). LGR4 was found to be preferentially expressed in osteoblasts and played a vital role in canonical Wnt signaling, thus regulating osteoblastogenesis and bone homeostasis (Zhang et al., 2021). MiR-193a-3p inhibited osteoblast differentiation through regulating LGR4/ATF4 signaling (Wang et al., 2018). Zhang, M. et al. reported that RSPO3-LGR4 system inhibited osteogenesis of human adipose-derived stem cells by negatively regulating ERK/FGF signalling (Zhang M. et al., 2017). The compressive force (CF) in alveolar bone led to the elevation of RANK and decrease of LGR4, thus inducing bone differentiation (Matsuike et al., 2018). LGR4 played an essentia role in the sequential development of molars by Wnt/β-catenin/LEF1 signaling (Yamakami et al., 2016). The silencing of LGR4 suppressed proliferation and osteogenic differentiation of stem cells from apical papillae (SCAPs) through inhibiting the Wnt/β-catenin pathway (Zhou et al., 2017). Arima, M. et al. reported that RSPO2-LGR4 accelerated osteoblastic differentiation by Wnt/β-catenin signaling in immature human periodontal ligament cells (Arima et al., 2019).

Further study indicated that there was a close correlation between LGR4 genotypes and bone mineral density (BMD), including the association between rs11029986 of LGR4 and total fat mass (TFM) (Yu et al., 2020). Additionally, researchers identified that a rare nonsense mutation within LGR4 gene (c.376C > T) was strongly associated with lower BMD and osteoporotic fractures by whole-genome sequencing of Icelandic individuals (Styrkarsdottir et al., 2013). Meanwhile, by the technology of next generation sequencing (NGS), Li, C. et al. showed that LGR4 was significantly differentially expressed between postmenopausal cases with impaired BMD and control group with normal values (Li C. et al., 2020). Moreover, a study also reported that LGR4-deficiency inhibited the differentiation of bone marrow mesenchymal stem cells (BMSCs), reduced bone mass, thus suppressing fracture healing (Sun et al., 2019). MiR-137 was correlated with an increased risk of fracture in patients with osteoporosis by targeting LGR4/ALP expression (Liu and Xu, 2018). In addition, a latest finding showed that the novel RANKL variant induced the expression of LGR4 by the GSK3-β signaling, thus suppressing the activity of NFATc1 and inhibiting osteoporosis (Ko et al., 2021). Shi, G. X. et al. identified that Rspo1/LGR4 could enhance osteogenesis by Wnt/β-catenin signaling. LGR4 might be a novel molecular protein in the transmission of mechanical stimuli to bone reorganization (Shi et al., 2017). Further research found that LGR4 induced the expression of pyruvate dehydrogenase kinase 1 (pdk1) via the canonical Wnt/β-catenin signaling. Loss-of-function experiments indicated that LGR4 deficiency resulted in decreased osteogenic effects together with aerobic glycolysis (Yang et al., 2021). The above studies revealed the important mechanisms of LGR4 in bone differentiation and development, indicating its great potential in the treatment of osteolysis diseases.

### LGR4 Gene in the Development of Heart, Liver, Kidney, Gonads, and Other Important Organs

Recent study indicated that Rspo3-LGR4 axis played a crucial role in heart development (Da Silva et al., 2018). LGR4 was found to be a molecular biomarker for cardiac progenitors (den Hartogh et al., 2016). In addition, study found that RSPO-LGR4/5-ZNRF3/RNF43 system regulated metabolic liver zonation by Wnt/β-catenin signalling (Planas-Paz et al., 2016). In contrast, Planas-Paz, L. et al. explored that LGR4/5-modulated WNT/β-Catenin signaling was dispensable for ductular reaction (DR) in biliary epithelial cells (BECs), while YAP and mTORC1 signaling were necessary for this process. LGR5 and AXIN2 were detected in hepatocytes to facilitate liver regeneration (Planas-Paz et al., 2019). More researches were needed on the important role of LGR4 gene in liver metabolism. Additionally, researchers proposed that the N-termini and 7TM domains of LGR5/LGR4 modulated WNT signaling in a ligand-dependent manner, while their C-termini and rhodopsin-like 7TM domains activated NF-κB signaling in a ligand-independent manner to control the survival of LGR5+ stem cells and intestinal crypts (Lai et al., 2020). Moreover, Dang, Y. et al. identified that the deficiency of LGR4 led to polycystic lesions and renal fibrosis by regulating Wnt/PCP signaling but not the TGF-β/Smad pathway (Dang et al., 2014). The serious renal hypoplasia was observed in LGR4 null mice (Kato et al., 2006). Conversely, Vidal, V. P. et al. explored that knockout of LGR4/5/6, the receptors of R-spondins, did not intervene with MET of nephron progenitor, revealing LGR-independent role in kidney development (Vidal et al., 2020). It was possible that the differences in mouse species and experimental conditions led to the differences in conclusion. A study revealed that high parathyroid hormone (PTH) elevated the expression of LGR4 and RANKL to facilitate vascular calcification (VC) by PTH1R/PKA activation (Carrillo-López et al., 2020). Luo, W. et al. identified that LGR4 promoted prostate development and stem cell differentiation by Wnt, Hedgehog and Notch1 signaling (Luo et al., 2013). The secretome from activation of stromal-androgen receptor (AR) maintained the basal state of epithelial cells by LGR4/β-Catenin/ΔNP63α signaling and did not induce the clonogenic growth of benign prostate hyperplasia (BPH) (Chauhan et al., 2020). In addition, researchers found that LGR4, not LGR5 was indispensable for the hematopoietic differentiation of human pluripotent stem cells (hPSCs) by regulating transforming growth factor beta (TGF-beta)-SMAD2/SMAD3 signaling, thus controlling mesoderm induction and hematopoietic development (Wang Y. et al., 2020).

**FIGURE 3:**
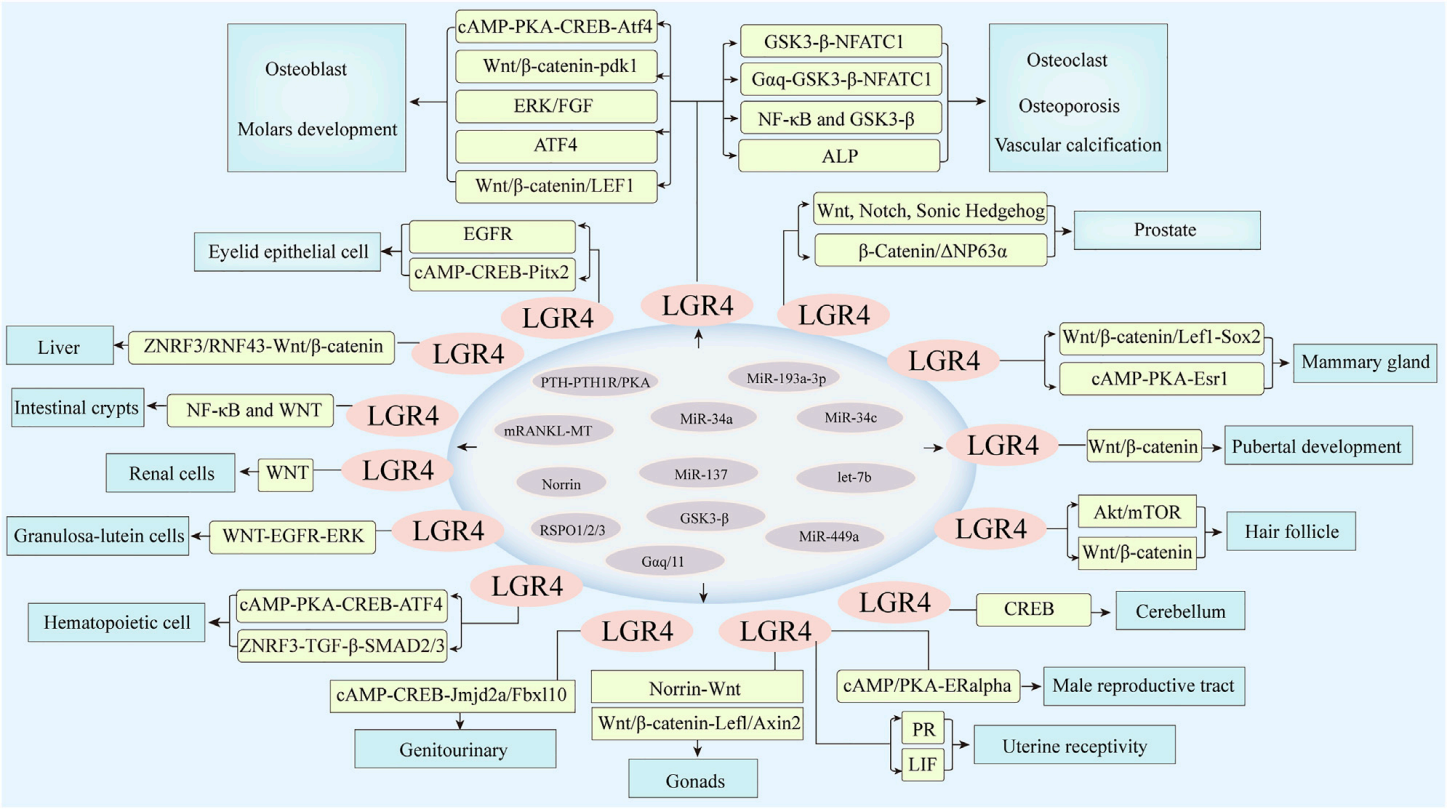
LGR4 was expressed in multiple tissues involved in the regulation of cell differentiation and tissue development. MicroRNAs including miR-34a, miR-34c, miR-193a-3p, miR-137, and miR-449a, let-7b could bind to the 3′ untranslated region of its target gene LGR4, resulting in its translational repression or degradation. Meanwhile, RSPO1/2/3, Norrin, and RANKL could act as ligands of LGR4. PTH-PTH1R/PKA, Gαq/11, GSK3-β could also activate LGR4, which then stimulated corresponding molecules and induced downstream signaling, such as Wnt/β-catenin, cAMP/PKA, Akt/mTOR, and so forth, thus regulating the differentiation of various organs.

In mammary gland, LGR4 induced the expression of Sox2 to facilitate mammary development via Wnt/β-catenin/Lef1 signaling pathway (Wang Y. et al., 2013). LGR4 could also promote corpus luteum maturation by WNT-mediated EGFR-ERK signaling, thus maintaining female fertility (Pan et al., 2014). Hsu, P. J. et al. explored that the LGR4 splice variant which encoded only the ectodomain of LGR4 (LGR4-ED) acted as an antagonist to suppress the LGR4/RSPO2/Norrin-mediated Wnt signaling thus controlling gonadal development (Hsu et al., 2014). Meanwhile, LGR4 could activate ERalpha by cAMP/PKA signaling to control the development of male reproductive tract (Li et al., 2010), and LGR4 inactivation led to the abnormal organization of it (Mendive et al., 2006). Hoshii, T. et al. explored that LGR4 knockout reduced the expression of estrogen receptor (ESR1), controlling elongation and differentiation of epididymal ducts (Hoshii et al., 2007). Further study explored that abnormal development of female gonads was observed in LGR4 (−/−) female mice. Rspo1/LGR4 was essential for ovarian somatic cell development via the Wnt/beta-catenin/Lefl/Axin2 signaling (Koizumi et al., 2015). In uterine receptivity, Kida, T. et al. explored that the phosphorylated PR was significantly reduced and persistent epithelial E2 receptor α was activated in LGR4 knockout mice, leading to impaired uterine receptivity (Kida et al., 2014). The reduced uterine glands and decidualization was observed in LGR4 knockout female mice by decreasing the secretion of LIF, implying the function of LGR4 in uterine gland development (Sone et al., 2013). Moreover, Gαq/11-coupled LGR4 promoted uterine receptivity by triggering PR signaling (de Oliveira et al., 2019). MiR-449a could promote caprine endometrial receptivity by targeting 3′-untranslated region of LGR4 (An et al., 2017). By using a bovine endometrial epithelial cell inflammation model and a mouse lipopolysaccharide-mediated endometritis model, the author confirmed that miR-34a/miR-193a-3p was upregulated by IL-1β and suppressed the level of the LGR4 3′UTR, which in turn amplified the inflammatory response through activating the phosphorylation of NF-κB p65 pathway, suggesting miR-34a/miR-193a-3p-LGR4 playing a pivotal role in endometritis (Ma et al., 2021; Yin et al., 2021). Furthermore, the gene of LGR4 modulated a WNT-NR5A2 signaling cascade facilitating the secretion, maturation and steroidogenesis of oviduct epithelial cells to safeguard the development and function of oviduct in mice (Tan et al., 2021). Other studies reported that akermanite elevated the expressions of integrinβ1, LGR4, LGR5, and LGR6, accompanied by triggering the Wnt/β-catenin pathway (Wang F. et al., 2020), thereby accelerating re-epithelialization in wound healing (Table 1 and Figure 3). Taken together, LGR4 was widely expressed in various tissues and played a fundamental role in modulating their development in a tissue-specific manner.

**TABLE 1:** Diverse roles of LGR4 in organ development.

Organ development	Signaling	Effect	References
Cerebellum	LGR4-Creb signaling	LGR4 (−/−) mice led to impairing long term depression	Guan et al. (2014)
Hair follicle	LGR4-Akt/mTOR signaling, Wnt/β-catenin signaling and decreasing BMP signaling	LGR4 promotes the normal hair cycle	Ren et al. (2020)
Facial organs	WNT9b:RSPO2-WNT/β-catenin	Wnt9b; Rspo2 double mutant mice displayed facial defects	Jin et al. (2020)
Pubertal development	LGR4-Wnt/β-catenin signaling	Mice deficient in LGR4 had delayed onset of puberty	Mancini et al. (2020)
Ocular cells	MicroRNA let-7b- LGR4; LGR4- cAMP-CREB- Pitx2; LGR4-EGFR	The antioxidant enzymes were decreased in LGR4 (−/−) mice	Jin et al. (2008); Weng et al. (2008); Wang et al. (2010); Zhu et al. (2015); Dong et al. (2016)
Liver	RSPO-LGR4/5-ZNRF3/RNF43- Wnt/β-catenin	Recombinant RSPO1 protein increased liver size	Planas-Paz et al. (2016)
Kidney	LGR4-WNT signaling; LGR4-cAMP-CREB-Jmjd2a/Fbxl10; MiR-34a- LGR4	LGR4 deficiency led to polycystic lesions and renal fibrosis	Dang et al. (2014); Yi et al. (2014); Hou et al. (2016)
Intestine	LGR5/4-NF-κB and WNT signaling	LGR5/LGR4 promoted the growth of intestinal crypts	Lai et al. (2020)
Vascular cells	PTH-PTH1R/PKA-LGR4	High PTH increases LGR4 thereby favouring vascular calcification	Carrillo-López et al. (2020)
Hematopoietic cell	LGR4-cAMP-PKA-CREB-ATF4; R-spondin1/R-spondin3/LGR4/ZNRF3-TGF-beta-SMAD2/SMAD3 signaling	LGR4^−/−^ fetuses displayed anemia, deletion of LGR4 limited hematopoietic differentiation	Song et al., 2008; Wang et al., 2020b
Osteoclast	LGR4-Gαq-GSK3-β-NFATC1; RSPO-LGR4-IQGAP1-Wnt/β-catenin; MiR-34c-LGR4-NF-Κb/GSK3-β; MiR-137-LGR4-ALP; RANKL-GSK3-β signaling-LGR4	LGR4 deficiency exhibit osteoclast hyperactivation	Carmon et al. (2014); Luo et al. (2016); Cong et al. (2017); Liu and Xu, (2018); Park et al. (2020); Jang et al. (2021); Ko et al. (2021)
Osteoblast	LGR4-cAMP-PKA-CREB-Atf4; LGR4-WNT/β-catenin; MiR-193a-3p-LGR4/ATF4; RSPO3-LGR4-ERK/FGF; RSPO1/2-LGR4-Wnt/β-catenin; LGR4-Wnt/β-catenin-pdk1/LEF1	Deletion of LGR4 results in a delay in osteoblast differentiation	Luo et al. (2009); Yamakami et al. (2016); Shi et al. (2017); Zhang et al. (2017a); Zhou et al. (2017); Wang et al. (2018); Arima et al. (2019); Yang et al. (2021); Zhang et al. (2021)
Gonads	LGR4-ED-LGR4/RSPO2/Norrin-Wnt; RSPO1/LGR4-Wnt/β-catenin- Lefl/Axin2; LGR4-WNT-EGFR-ERK signaling	LGR4-ED acted as an antagonist controlling gonadal development	Hsu et al. (2014); Pan et al. (2014); Koizumi et al. (2015)
Mammary gland	LGR4-Wnt/β-catenin/Lef1-Sox2; Rspo1-LGR4-cAMP-PKA-Esr1	LGR4 (−/−) mice had delayed ductal development	Wang et al. (2013c); Geng et al. (2020)
Prostate	LGR4-Wnt, Notch, Sonic Hedgehog signaling; LGR4/β-Catenin/ΔNP63α; LGR4-cAMP/PKA-ERalpha	LGR4 loss blocked differentiation of prostate cells	Li et al. (2010); Luo et al. (2013); Chauhan et al. (2020)
Uterine	LGR4-PR/LIF; Gαq/11-LGR4-PR; MiR-449a-LGR4; MiR-34a/miR-193a-3p-LGR4-NF-κB	LGR4 KO down-regulated progesterone signaling, affecting uterine receptivity and led to endometritis	Sone et al. (2013); Kida et al. (2014); An et al. (2017); de Oliveira et al. (2019); Ma et al. (2021); Yin et al. (2021)
Oviduct	LGR4-WNT-NR5A2 signaling	The loss of LGR4 ultimately impaired the epithelial secretion	Tan et al. (2021)

## Regulation of LGR4 Gene in Immune-Related Diseases

Cancer immunotherapy has demonstrated marvelous efficacy in clinical trials targeting negative immune checkpoint mediators including CTLA-4 and PD-1 (Pardoll, 2012). Tumor-associated macrophages (TAMs) constituted the major leukocytic infiltrate and could be polarized to a proinflammatory “M1” or a immunosuppressive “M2” phenotype by tumor-derived chemokines or cytokines (Mantovani et al., 2004). Tan, B. et al. demonstrated that Rspo-LGR4 axis functioned as a novel pathway aggravating M2-like macrophage polarization through noncanonical Erk/Stat3 signaling by recruiting IQGAP1 and MEK1/2, thus maintaining protumoral TAMs, promoting tumor progression and enhancing the resistance of Lewis lung carcinoma (LLC) cells to anti-PD1 treatment. Accordingly, LGR4 deficiency only in macrophages was able to activate both macrophage-mediated innate and T-cell–mediated adaptive antitumor immune responses, thus overcoming resistance to checkpoint blockade therapy, which gave this axis great potential as a promising therapeutic target in macrophage-targeting strategies (Tan et al., 2018). Moreover, LGR4 negatively modulated CD14 transcriptional activation and inhibited TLR2/4-associated immune response via cAMP-PKA-CREB signaling (Du et al., 2013). Liu, S. et al. identified that LGR4 prevented intestinal inflammation by modulation of the Wnt/β-catenin signaling (Liu S. et al., 2013). Another study explored that the expression of LGR4 was reduced in traumatic osteoarthritis. Overexpression of LGR4 could suppress the joint inflammation by inhibiting NF-κB pathway (Ge et al., 2019). MiR-34a and miR-34c enhanced inflammatory response and delayed chronic wound healing of venous ulcers by direct targeting LGR4. MiR-34-LGR4 axis reduced GSK-3β-induced phosphorylation of p65 at Ser468, while enhancing phosphorylation at Ser536, activating NF-kB signaling, thus regulating inflammatory response of keratinocyte (Wu et al., 2020). Zhang, N. et al. showed that LGR4 functioned as a vesicular stomatitis virus (VSV)-specific host factor enhancing VSV infection, LGR4 knockdown reduced the levels of VSV(Zhang N. et al., 2017). Furthermore, LGR4 was related to the infection of severe acute respiratory syndrome coronavirus (SARS-CoV) (Liu H.-L. et al., 2020) (Table 2), and may be a potential gene target for therapy. Together these data pointed towards a key role for LGR4 in immune-related diseases, though further studies were needed to better establish this, especially in the field of tumor immunotherapy.

**TABLE 2:** The functions of LGR4 in immune-related diseases.

Diseases	Signaling	Effect	References
Lung carcinoma associated macrophage	Rspo/LGR4/Erk/Stat3-enhanced macrophage M2 polarization	Blocking Rspo-LGR4 signaling overcame lung carcinoma resistance and suppressed tumor growth	Tan et al. (2018)
Macrophage in innate immunity	LGR4-cAMP-PKA-CREB signaling-CD14	LGR4-deficiency led to increased immune response	Du et al. (2013)
Intestinal inflammation	LGR4-Wnt/β-catenin signaling	LGR4 (−/−) mice exhibited stronger intestinal inflammation	Liu et al. (2013b)
Traumatic osteoarthritis	LGR4-NF-κB signaling	Upregulation of LGR4 expression can inhibit the secretion of the inflammatory factors	Ge et al. (2019)
Keratinocyte in venous ulcers	MiR-34-LGR4-GSK-3β-induced p65 phosphorylation-NF-κB signaling	Knockout of LGR4 impaired wound closure with enhanced inflammation	Wu et al. (2020)
Vesicular stomatitis virus	Vesicular stomatitis virus-LGR4	LGR4 knockdown suppressed VSV infection	Zhang et al. (2017b)

## The Role of LGR4 in Metabolic Diseases

The expression of LGR4 was detected by *in situ* hybridization assay and was elevated in hypothalamic energy homeostatic areas and co-localizated with some energy homeostatic neurons suggesting that it may regulate energy homeostasis (Van Schoore et al., 2005). In line with this, Otsuka, A. et al. showed that R-spondin1-LGR4 suppressed appetite of mice by upregulating Pomc gene expression. The suppressed food intake was not observed in LGR4 knockdown mice (Otsuka et al., 2019). Rspo1/Rspo3/LGR4 forming novel system to regulate feeding behavior (Li et al., 2014). Moreover, LGR4 ablation promoted the energy switch from glucose to fatty acid by activating Ampk/Sirt1/Pgc1α pathway (Sun et al., 2015). Importantly, the LGR4 A750T variant was investigated by Sanger sequencing, and the result found that it was correlated with central obesity (Zou et al., 2017). In addition, Shi et al. demonstrated that genetic polymorphisms of the LGR4 gene were related to bone and obesity phenotypes in Chinese nuclear families with female children (Shi et al., 2021). Furthermore, LGR4 was found to modulate energy balance and body weight through regulating the translation of white fat into brown fat (Wang J. et al., 2013). A recent study reported that RSPO1/LGR4 axis was involved in obesity-related renal fibrosis through promoting Wnt/β-catenin signaling pathway, providing a potential therapeutic target for the obesity-related chronic kidney disease (CKD) (Su et al., 2021). Likewise, LGR4, as a adipocytokine, was closely related to the progression of diabetes and hypertension (Li B. et al., 2019). Notably, Wang, J. et al. identified that LGR4 elevated mineralocorticoid receptor (MR) expression by cAMP/protein kinase A pathway to improve aldosterone responsiveness and maintain electrolyte homeostasis (Wang et al., 2012). Meanwhile, Rspo1/Rspo3-LGR4 signaling attenuated cholesterol synthesis in hepatocytes by activating the phosphorylation of AMPKα and suppressing SREBP2 nuclear translocation (Liu S. et al., 2020) (Table 3). Hence, the function of LGR4 in energy metabolism was being broadly studied, a better understanding of the molecular mechanism underlying various metabolic pathways involved by LGR4 will help in future development of new treatments for metabolic diseases.

**TABLE 3:** Regulatory effects of LGR4 gene on metabolic diseases.

Pathway	Effect	References
LGR4-cAMP/PKA	LGR4 KO mice had aldosterone resistance	Wang et al. (2012)
Rspo1/Rspo3/LGR4	Injection of Rspo1 or Rspo3 inhibited food intake	Li et al. (2014)
R-spondin1-LGR4-Pomc	LGR4 KO mice didn’t exhibit a suppressed appetite	Otsuka et al. (2019)
LGR4-Ampk/Sirt1/Pgc1α pathway	LGR4 ablation enhanced fuel shift	Sun et al. (2015)
Rspo1/Rspo3-LGR4-AMPKα-SREBP2 pathway	Rspo1/Rspo3-LGR4 signaling suppresses cholesterol synthesis	Liu et al. (2020b)

## The Role of LGR4 in Oxidative Stress Damage

Oxidative stress exerted an increased impact on pathophysiology of osteoporosis. However, the correlation between LGR4 and oxidative stress remained unknown. Pawaputanon Na et al. explored that the treatment of hydrogen peroxide decreased the expression of LGR4 in osteoblastic cells (Pawaputanon Na Mahasarakham et al., 2017). Rspo1-LGR4 axis protected hepatocytes against acute injury by suppressing NF-κB-p65 via Wnt3a/β-catenin pathway (Li Z. et al., 2019). Of note, Liu, S. et al. reported that Rspo3-LGR4 system protected hepatocytes from dimethyloxalylglycine (DMOG)-caused hypoxia/reoxygenation (H/R) damage by Wnt3a/β-catenin (Liu et al., 2018). LGR4 also controlled mitochondrial function and oxidative stress by activating ERK signaling, thus protecting myocardium against ischemia-reperfusion (I/R) damage (Chen T. et al., 2021). Additionally, Singla, B. et al. showed that RSPO2-LGR4 interaction reduced lymphangiogenesis by inhibiting PI3K-AKT-eNOS and Wnt-β-catenin pathway. LGR4 silencing could block this process and facilitate cholesterol drainage from atherosclerotic arteries (Singla et al., 2020). Conversely, Huang, C. K. et al. showed that LGR4 could induce proinflammatory responses in myocardial infarction (MI) by elevating the expression of AP-1 via CREB-regulated c-Fos, Fosl1, and Fosb activation. Knockout of LGR4 could mitigate ischemic injury (Huang et al., 2020). Study uncovered that radiation therapy elevated the expression of Rspo1 and LGR4 in bone mesenchymal stem cells (BMSCs). Exogenous Rspo1 reduced radiation-induced bone damage by Rspo1-LGR4-mTOR-autophagy signaling (Chen X. et al., 2021) (Table 4). Therefore, LGR4 was involved in oxidative stress and cellular damage, a deeper exploration of its mechanisms was still required. From a broader and longer-term perspective, these investigations may slow or even reverse the onset of stress injuries by targeting LGR4 gene.

**TABLE 4:** Increased impacts of LGR4 gene on oxidative stress response.

Pathway	Effect	References
Rspo1-LGR4-Wnt3a/β-catenin-NF-κB-p65	LGR4 protected hepatocytes from injury	Liu et al. (2018); Li et al. (2019b)
LGR4-ERK signaling-oxidative stress	LGR4 protected cardiomyocyte against I/R	Chen et al. (2021a)
RSPO2-LGR4-PI3K-AKT-Enos/Wnt-β-catenin	LGR4 silencing promoted lymphangiogenesis	Singla et al. (2020)
LGR4-CREB-mediated c-Fos/Fosl1/Fosb/AP-1	LGR4 knockout infarcts had reduced inflammatory	Huang et al. (2020)
Rspo1-LGR4-mTOR-autophagy	Exogenous Rspo1-LGR4 alleviated radiation-induced bone loss	Chen et al. (2021b)

## Suggested Roles of LGR4 in Carcinomas

**FIGURE 4:**
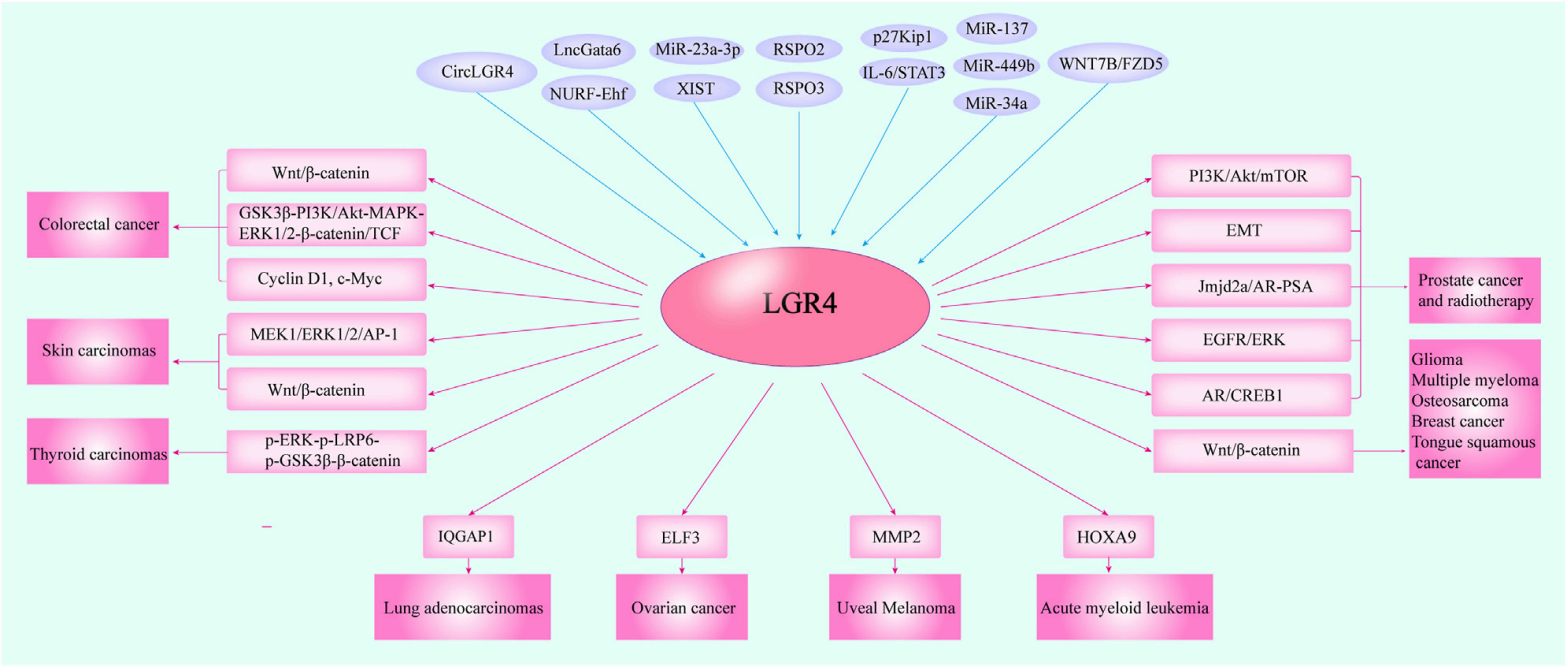
The cross-talking of LGR4 with other molecules in multiple carcinomas. Several studies indicated that LGR4 was overexpressed in cancer tissues. The molecules including circLGR4, lncGata6-NURF-Ehf, XIST/miR-23a-3p, miR-137, miR-449b, miR-34a, IL-6/STAT3, p27Kip1, WNT7B/FZD5, and RSPO2/3 could function as upstream regulators of LGR4 and mediate its expression. Then, LGR4 could modulate the proteins such as IQGAP1, ELF3, MMP2, and HOXA9 to control tumor progression and trigger the Wnt/β-catenin, MEK1/ERK1/2/AP-1, PI3K/Akt pathways to promote the initiation and metastasis of a variety of malignancies.

The gene of LGR4 emerged as a critical player in regulation of tumor growth and progression (Gong et al., 2015; Liang et al., 2015). Zhu, Y. B. et al. identified that LGR4 induced Wnt/β-catenin signaling to promote cancer cell growth and migration (Zhu et al., 2013). Accordingly, Kang, Y. E. et al. demonstrated that R-spondin 2 and LGR4 were overexpressed in thyroid cancer. The upregulated LGR4 enhanced cell proliferation and migration by inducing the phosphorylation of ERK and GSK3β and activating β-catenin pathway (Kang et al., 2017). The expression of LGR4 was elevated after prostate cancer radiotherapy. LGR4 ablation inhibited AR/CREB1 expression, promoted γH2A.X staining and reduced tumor growth (Liang et al., 2021). Moreover, LGR4 was overexpressed in human prostate cancer and correlated with shorter disease-free survival. The knockdown of LGR4 inhibited cell migration and reversed EMT by elevating the expression of E-cadherin (Luo et al., 2017). Also, Zhang, J. et al. revealed that LGR4 promoted Jmjd2a/AR signaling to enhance AR binding to PSA promoter, thus contributing to inducing prostate tumorigenesis (Zhang et al., 2016). LGR4 may also facilitate the growth of prostate cancer via the PI3K/Akt/mTOR signaling (Liang et al., 2015). Furthermore, hypoxia exposure downregulated the expression of miR-137, which targeted LGR4 and prevent the migration and EMT of prostate cancer by inhibiting EGFR/ERK signaling (Zhang et al., 2020). When a hypomorphic mutation of LGR4 was observed in chronic myelogenous leukaemia (CML) stem cells, it induced inadequate disease-initiating capacity of CML cells in mice (Naka et al., 2020). With regard to acute myeloid leukemia (AML), Salik, B. et al. also identified that LGR4 was upregulated and cooperated with HOXA9 in AML. RSPO3-LGR4 interaction enhanced proliferation and self-renewal of AML blasts (Salik et al., 2020). Study identified that LGR4 expression was associated with poor prognosis in breast cancer. The down-regulation of LGR4 inhibited Wnt signaling and epithelial-mesenchymal transition (EMT), suppressing mammary tumorigenesis (Yue et al., 2018). Additionally, Yue et al. uncovered a Wnt-independent LGR4-EGFR signaling axis enhancing breast cancer cell metastasis with broad implications for the targeted therapy of breast cancer (Yue et al., 2021). Importantly, Gao, Y. et al. identified that the short hairpin RNA of LGR4 could suppress the invasion and metastasis of HeLa cells (Gao et al., 2009). In addition, Berti FCB. et al. found that for HPV16-cervical squamous carcinoma (CESC), eleven miRNAs were shared by XIST/LGR4 and XIST/ZNF81 lncRNA-mRNA co-expressed pairs, implying an increased effect on their ultimate biological effect. Moreover, XIST/miR-23a-3p/LGR4 had a remarkable impact on the overall survival of HPV18- and HPV16-CESC patients (Berti et al., 2021). LGR4/ELF3 axis could also enhance the epithelial phenotype of serous ovarian cancer and was mediated by WNT7B/FZD5 interaction (Wang Z. et al., 2020). By analyzing 122 serous ovarian cancer tissues and 41 paired para-carcinoma tissues, Zeng, Z. et al. showed that the expression of LGR4 was elevated in serous ovarian cancer and it could be used as an independent prognostic predictor (Zeng et al., 2020). Specifically, a study revealed that LGR4 was upregulated in colorectal cancer (CRC) and enhanced tumor metastasis by PI3K/Akt and MAPK/ERK1/2-mediated β-catenin/TCF signaling (Wu et al., 2013). Gao, Y. et al. demonstrated that enhanced expression of LGR4 caused by p27Kip1 deficiency promoted metastasis of colon cancer cells (Gao et al., 2006). Currently, CircLGR4 was highly expressed in colorectal tumors. CircLGR4-derived peptide activated LGR4 and then promoted Wnt/β-catenin signaling, thus driving colorectal tumorigenesis (Zhi et al., 2019). Additionally, LncGata6 recruited the NURF complex onto the promoter of Ehf to enhance its transcription, which elevated the expression of LGR4/5 to activate Wnt signaling, thus promoting the progression of colorectal cancer (Zhu et al., 2018). Wang, Y. et al. showed that the expression of LGR4 was elevated in uveal melanoma cells. MiR-34a negatively controlled the expression level of LGR4, thus downregulating the markers of the EMT and MMP2, thereby impacting the aggressiveness of uveal melanoma (Hou et al., 2019). Study reported that LGR4 was considered as an independent prognostic marker for patients with non-small cell lung cancer (NSCLC) (Li R. et al., 2020). MiR-449b as a tumor suppressor prevented the proliferation of NSCLC by downregulating LGR4 (Yang et al., 2018) and LGR4 was perceived as a high-risk immune gene in NSCLC (Sun et al., 2020). The aberrant activation of RSPO3-LGR4-IQGAP1 system promoted tumor aggressiveness in Keap1-deficient lung adenocarcinomas. Knockdown of LGR4 led to reduction in cell proliferation (Gong et al., 2015). Additionally, Zhang, L. et al. showed that Rspo2-LGR4 system promoted the growth and migration of tongue squamous cell carcinoma (TSCC). It potentiated β-catenin pathway by enhancing phosphorylation of LRP6, while reducing phosphorylation of GSK-3β, contributing to subsequent upregulation of TCF-1 and its downstream genes CD44, c-Myc, and Cyclin D1, thus facilitating the progression of TSCC (Zhang et al., 2019). LGR4 could also promote the proliferation of glioma by activating Wnt/β-catenin signaling (Yu et al., 2013). Stat3 could elevate LGR4 expression by binding to LGR4 promoter, thereby regulating osteosarcoma progression (Liu J. et al., 2013). Furthermore, van Andel, H. et al. identified that R-spondin/LGR4 axis promoted multiple myeloma (MM) by activating aberrant Wnt/β-catenin signaling (van Andel et al., 2017). LGR4 acted as an essential positive factor for inducing skin tumorigenesis by activating MEK1/ERK1/2/AP-1 and Wnt/β-catenin pathways (Xu et al., 2016). Conversely, Souza, S. M. et al. detected that LGR4 was expressed in a larger number of cells in normal gastric mucosa than in primary gastric carcinomas and not specific to gastric cancer cells, predominantly affecting the expression of β-catenin in membrane-complex but rarely in nucleus, suggesting a controversial function of LGR4, and which was positively correlated with cell proliferation but inversely related to cancer progression (Souza et al., 2019) (Table 5 and Figure 4). Due to various carcinogenic factors, multiple cellular microenvironment and a variety of cell types, the complicated functions of LGR4 in carcinogenesis needed further exploration. Therefore, given the multitude of indications for the relevant oncogenic role of LGR4, it remained valuable to further investigate the clinical potential of anti-LGR4 monoclonal antibodies specifically in cancer patients that harbored LGR4 alterations, which would hopefully provide more insight beneficial to the development of novel treatment strategies against LGR4 driven cancer.

**TABLE 5:** The gene of LGR4 involving in the process of multiple tumors.

Cancer subtypes	Pathway	Effect	References
Skin carcinoma	LGR4-MEK1/ERK1/2/AP-1 and Wnt/β-catenin pathways	LGR4 was crucial for skin carcinogenesis	Xu et al. (2016)
Glioma	LGR4-Wnt/β-catenin	LGR4 overexpression promoted cell proliferation	Yu et al. (2013)
Uveal melanoma	MiR-34a-LGR4-MMP2	Knockdown of LGR4 attenuated the aggressiveness	Hou et al. (2019)
Tongue carcinoma	Rspo2-LGR4-Wnt/β-catenin	Elevated LGR4 promoted growth	Zhang et al. (2019)
Thyroid carcinomas	R-spondin2-LGR4-p-ERK-p-LRP6–p-GSK3β-β-catenin	Elevated expression of LGR4 promoted proliferation and migration	Kang et al. (2017)
Lung cancer	RSPO3-LGR4- IQGAP1, MiR-449b-LGR4	Knockdown of LGR4 decreased tumor growth	Gong et al. (2015); Yang et al. (2018)
Colon cancer	p27Kip1- LGR4, CircLGR4-LGR4-Wnt/β-catenin, LGR4-GSK3β-PI3K/Akt-MAPK-ERK1/2-catenin/TCF-Cyclin D1/c-Myc, LncGata6-NURF-Ehf-LGR4/5-Wnt	LGR4 expression was associated with colorectal tumorigenesis	Gao et al. (2006); Wu et al. (2013); Zhu et al. (2018); Zhi et al. (2019)
Acute myeloid leukemia	RSPO3-LGR4-HOXA9	RSPO3-LGR4 interaction promoted proliferation	Salik et al. (2020)
Multiple myeloma	IL-6/STAT3-LGR4/R-spondin- Wnt/β-catenin	LGR4 expression was driven by IL-6/STAT3 signaling and allowed MM cells to hijack R-spondins	van Andel et al. (2017)
Osteosarcoma	Stat3-LGR4	Overexpression of Stat3 promoted LGR4 expression	Liu et al. (2013a)
Breast cancer	LGR4- Wnt/β-catenin signaling, LGR4-EGFR signaling	LGR4 down-regulation decreased tumor growth and lung metastasis	Zhu et al. (2013); Yue et al. (2018); Yue et al. (2021)
Prostate cancer	LGR4-EMT, LGR4-Jmjd2a/AR signaling-PSA, MiR-137-LGR4-EGFR/ERK, LGR4-AR/CREB1 expression, LGR4-PI3K/Akt/mTOR	LGR4 knockdown impaired cell migration	Liang et al. (2015); Zhang et al. (2016); Luo et al. (2017); Zhang et al. (2020); Liang et al. (2021)
Ovarian cancer	WNT7B/FZD5-LGR4/ELF3 axis	LGR4 overexpression enhanced tumorisphere formation capacity	Wang et al. (2020c)
Cervical cancer	XIST/LGR4, XIST/miR-23a-3p/LGR4	—	Berti et al. (2021)

## Discussion and Future Directions

In general, a growing body of evidence indicated that LGR4 was widely expressed in diverse tissues from the early embryogenesis to adulthood, participated in the differentiation and development of various organs, involved in immune-related diseases, metabolic diseases as well as oxidative stress damage and contributed to multiple cancer progression. The repertoire of the physiological and pathological roles of LGR4 in numerous cellular processes provided a systematic and comprehensive understanding of its functional characteristics, thereby offering a novel diagnostic biomarker and therapeutic target for a range of diseases.

The interaction between LGR4 and its ligands including RSPOs, Norrin, RANKL, and could activate the downstream Wnt pathway and other G protein-associated pathways. Several researches implicated that inhibitors or antagonists of the RSPOs/LGR4/Wnt/β-catenin axis could suppress tumor metastasis and recurrence. Moreover, blockage of the LGR4 signaling would result in a decreased population and impaired the migration ability of cancer stem cells, which may lay a solid theoretical basis for the development of small molecule blockers and antagonizing antibodies to suppress LGR4 pathway.

Additionally, with regard to the LGR4/cAMP/PKA signaling, the endogenous ligands that activated LGR4 were yet to be elucidated clearly. Meanwhile, the function of LGR4 through its newly discovered ligand RANKL was mainly detected in maintaining homeostasis of bone tissue, whereas their involvement in malignancies was rarely explored. Hence, further detailed mechanism investigation was crucial for representing the spatiotemporal profile of LGR4 and might open up a new avenue for molecular targeted therapy for tumors and other diseases.
